# In Memoriam: Professor Plinio ROSSI (1929–2025)

**DOI:** 10.5334/jbsr.4176

**Published:** 2025-12-15

**Authors:** Robert F. Dondelinger

**Affiliations:** 1 University of Liège, Liège, Belgium

Professor Plinio ROSSI was born in the small town of Santi Cosma e Damiano in Central Italy on June 4, 1929. His father was a medical doctor, and his mother a grammar schoolteacher. During the Second World War, their parental home in Formia was bombed to the ground by a German raid. Plinio and his elder brother lived an odyssey on shoestrings during the war without benefitting from regular school teaching. Following a nine‑month crash course covering the syllabi of three years of secondary school, Plinio obtained a secondary school certificate at the end of June 1946 at the age of 17. In the fall, he enrolled in medical school at the University of Rome. He graduated in 1952, earning the highest obtainable grade. Plinio tried to enter specialty training in radiology in Florence, but admission was refused due to a late application. Plinio followed the specialty course in respiratory disease and attended the division of surgical semiotics as a volunteer assistant at Careggi Hospital, a branch of the University of Florence. In 1953, he returned to Formia and worked at the newly built Ospedale Dono Svizzero. Plinio learned many aspects of medicine and surgery without getting paid. On May 14, 1954, he left for the USA with a student visiting visa and 140 US$ in his pocket. Plinio started a one‑year internship at Martland Medical Center in Newark, New Jersey, earning 80 US$ a week for a workload of 90 h per week as an all‑round doctor. Plinio tried to choose radiology again as a medical specialty. As an Italian immigrant, he was forced to accept the only available offer from the public Queens General Hospital for a three‑year radiology residency, starting on July 1, 1955. There was no official radiology teaching in the department; the residents had to make the most of any learning opportunity that arose. Plinio picked up them all, and soon he became the reference radiologist for general and cerebral diagnostic arteriography. At the end, Plinio had performed about 400 translumbar aortographies. The hospital director encouraged Plinio to change his visa. Plinio obtained his first immigrant passport and continued his training until autumn 1958. He registered at New York University and followed nine‑month compulsory studies for foreign‑graduated doctors before passing examinations for the board certification in radiology at the end of 1959. Plinio had learned everything by himself and started to make a name for himself as a vascular radiologist in NYC. He received an offer from the Catholic non‑for‑profit St. Vincent’s Hospital in the bustling heart of Manhattan. On January 1, 1960, Doctor Rossi officially started his career as a vascular diagnostic radiologist and soon after as an interventional radiologist. Besides conventional diagnostic radiology, Plinio committed himself day and night to peripheral, visceral, neuro‑, and coronary angiography, which contributed to his promotion as the head of the new angiodynamics division at St. Vincent. Plinio started publishing his personal innovative experience in angiography at the rate of 10 papers per year for the next six years. The increasing fame of Doctor Rossi prompted the chairman of Cornell University to invite him to lecture as a clinical professor of radiology at New York Hospital. After his marriage, Plinio opted for a radical change. He moved back to Rome in 1970 but continued commuting between the old and new worlds for the next couple of years. On his return to Italy, Plinio obtained an Italian specialization in radiology; he focused on examinations as a department chairman and an academic teacher. Plinio started clinical angiography at San Filippo Neri Hospital in Rome. Italy still lacked a professional update in angiography in these years. In 1972, Plinio set up in Rome the first international course in thoracic and abdominal vascular radiology (CARVAT), which stood for many years as the finest biennial teaching of advances in interventional angiography offered in Europe. Plinio won the competitive examination as a tenured assistant professor of radiology at the University La Sapienza in Rome. The next year, Plinio co‑founded the European Society of Cardiovascular Radiology (ESCVR), which became the European Society of Cardiovascular and Interventional Radiology (ESCVR) in 1975 and evolved into the Cardiovascular and Interventional Radiological Society of Europe (CIRSE) in 1985, merging with the European College of Angiography (ECA) set up by the Scandinavian angiographers. In the USA, he co‑founded the Society of Cardiovascular Radiology, which became the Society of Interventional Radiology (SIR) in 1973, and in Italy, he created a section devoted to vascular radiology within the Italian Society of Medical Radiology (SIRM). Plinio was the first to introduce selective arterial embolization techniques in Italy, notably in gastrointestinal and posttraumatic bleeds or in oncology. Plinio performed the first transluminal angioplasty in Italy in 1974, using a Porstmann caged balloon. Plinio joined the private Casa di Cura Villa Margherita facility and was the first in Italy to develop dynamic contrast‑enhanced angio‑CT. In 1983, Plinio was nominated as an associate professor at the University of Rome, and in 1986, he became a full professor of radiology at the University of Milan. Plinio continued commuting between Milan and Rome for several years. In 1990, he joined the founders of the European Society of Gastrointestinal Radiology (ESGR). He was instrumental in broadening the scope of the Society beyond barium studies, encompassing abdominal cross‑section imaging and intervention. In 1994, Plinio advised changing the name of the Society to European Society of Gastrointestinal and Abdominal Radiology (ESGAR). In 1991, Plinio was called back to Rome to take up the position of the third chair of radiology at La Sapienza and head of the division of interventional radiology at Policlinico Umberto I. In 1992, when he was still in Milan, Plinio performed the first transjugular intrahepatic portosystemic stent shunt (TIPSS) in Italy. Professor Rossi earned numerous distinctions, notably gold medals from nine scientific radiological societies. Plinio was an enthusiastic traveler, exchanging experiences with other researchers around the world, an indefatigable teacher, and a prolific writer, totaling about 400 publications. In 1990, I edited the first comprehensive textbook in interventional radiology in Europe in the English language, inviting Plinio as a co‑editor. Plinio retired from academic life in 2004, but he continued performing angiographic and interventional procedures in private practice. Plinio passed away on September 14, 2025. He is survived by his wife, Maddalena, and their children, Alessandra and Adriano. Professor Plinio Rossi is a unique luminary in angiography and intervention and an example for the upcoming generations of radiologists. Plinio was above all a medical doctor. Having spent his entire life on the edge of progress in intervention, he advised the radiologists ‘to act more like doctors and being more humane’.

**Figure:**
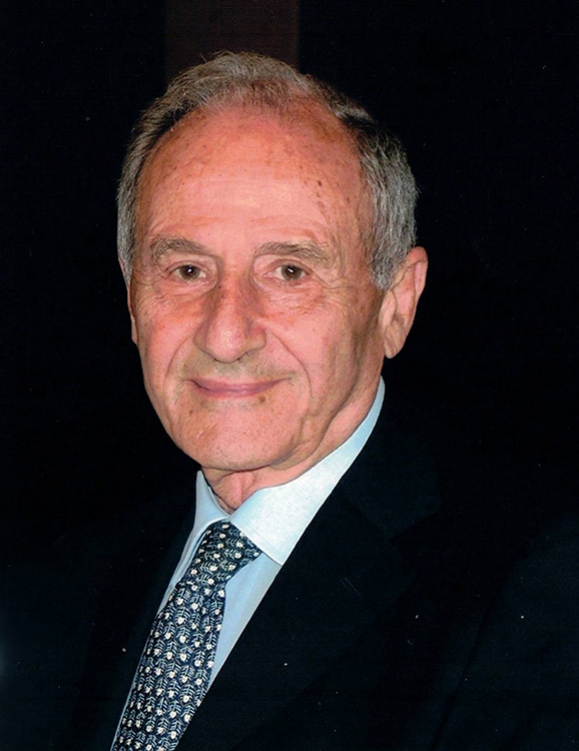
Professor Plinio Rossi

